# Three pillars for ensuring public access and integrity of chemical databases powering cheminformatics

**DOI:** 10.1186/s13321-025-00983-9

**Published:** 2025-03-28

**Authors:** Antony J. Williams, Ann M. Richard

**Affiliations:** Center for Computational Toxicology and Exposure, Office of Research and Development, U.S. Environmental Protection Agency, Research Triangle Park, Durham, NC 27711 USA

## Introduction

Since the inception of the Internet, public databases disseminating chemistry data to the community have proliferated and helped to support and encourage a burgeoning interest in cheminformatics. This has been supported by a shift in open science, exemplified by Open Data, Open Source, and Open Standards (ODOSOS) for chemistry [1], as well as by the increasing sophistication and availability of free and open source computational, machine-learning, and artificial intelligence approaches for mining and modeling chemical structure associated data.

The authors of this perspective have been engaged in using cheminformatics to distribute chemistry data to the community for over two decades. Our combined careers have had us apply cheminformatics in a Fortune 500 industrial company, in a commercial software company, in chemistry publishing, and in the government. As a result, we have experienced the challenges of both building and distributing chemistry data. While separately engaged in building publicly available chemical databases—namely, ChemSpider [2] and the U.S. Environmental Agency’s (EPA) DSSTox [3], over the past decade we have combined our efforts as colleagues within the EPA to institute automated and manual quality curation procedures, while expanding the reach and public availability of chemical-indexed information to a wide range of potential users via EPA’s CompTox Chemicals Dashboard (CCD) [4]. PubChem [5], ChEMBL [6], and many others have also been major contributors to the wealth of chemically indexed data available to the community, spanning a wide range of domains of potential relevance to industry, researchers, and regulatory agencies across the globe. In the remainder of this short perspective, we present what we believe are three chemical data and quality pillars that are essential to the continued growth and scientific impact of the cheminformatics field.

Pillar 1: Government funding and public support for structure-indexed, searchable, downloadable chemical databases

Within the U.S., across Europe and, to a lesser extent, in other nations, the role of government funding for the creation, hosting, and maintenance of large, publicly available chemical databases cannot be overstated. This financial support is generally through direct funding of multi-year research programs in US government agencies, as in the case of the National Institutes of Health’s (NIH) PubChem and EPA’s DSSTox and Dashboard, or indirectly, through grants and research funding to universities. Within the European Union (EU), regulatory bodies, such as the European Chemicals Agency (ECHA) and the European Food Safety Authority (EFSA) each provide public access to chemical databases. Increasingly over the past 2 decades, US government research agencies and institutes (such as EPA and NIH) have been mandated to meet high standards of public transparency, which includes scientific publications, Internet distribution, and open data mandates. Unlike industry and commercial chemical data stores, which are most often domain-limited and sequestered as intellectual capital, government chemical data records compiled for regulatory and research purposes can span broad chemical domains (e.g., drugs, pesticides, industrial, cosmetics, consumer products, etc.) and such data are mandated for public release whenever possible. EPA’s ToxCast [7] and the multi-federal agency Tox21 [8] high-throughput screening (HTS) programs are 2 prominent examples, with quality chemical curation for thousands of chemicals handled within EPA’s DSSTox database program, and all chemically associated HTS activity data publicly released in structure-searchable and downloadable form through EPA’s CCD and the NIH’s PubChem websites.

Securing adequate funding and resources for data curation to establish and ensure accurate chemical identifier and associated data linkages in new and historical records is a persistent challenge, but pales by comparison to the cost of generating new chemical data. Curation is often perceived as a low-priority task compared to research and development, but the latter enterprise is undermined from the start by lack of adequate attention to quality chemical curation (Pillar 3). We advocate for transparency and adoption of minimum quality curation standards, and ongoing support and investment by government agencies to not only ensure the long-term sustainability and value of chemical data resources, but to keep abreast of the continued expansion of chemistry data on the Internet.

Pillar 2: clear data licensing, provenance, and the need for FAIRness

The exchange and reuse of data between databases often leads to propagation of errors. The practice of mixing and aggregating content from various sources also makes it difficult to trace the origin of data (provenance). This lack of clarity can hinder verification and attribution, making it challenging to assess the reliability and quality of data. This is particularly problematic when the original source of the data is unclear. As an example, PubChem is a widely used database that aggregates user-deposited content and employs automated, source-weighted algorithms for assessing quality chemical structure associations. It does not, however, employ manual curation review except indirectly (i.e., when source content is curated). As it is often the source of chemical data for other databases, this can lead to a cycle of data propagation, crosstalk and error amplification as was the experience of one of the authors (AJW) when PubChem data were registered into ChemSpider [2]. In contrast, ChEMBL's manual curation process involves expert scientists meticulously extracting and standardizing bioactivity data, including chemical structures, target information, and assay details, directly from the primary scientific literature thereby ensuring high quality and consistency by manually drawing chemical structures, mapping to relevant targets, and annotating key data points, rather than solely relying on automated data mining techniques.

In addition, determining whether a collection of data is copyrightable can be difficult. Whereas individual data points, such as a melting point or connection table, are generally not copyrightable, a collection of chemical structures, identifiers, and experimental parameters might be considered intellectual property. This uncertainty complicates data sharing and reuse. Many scientists downloading data from public databases are likely unaware of potential licensing limitations or the importance of attribution, which can lead to unintentional misuse or misrepresentation of data. Conflicting licenses between different databases can also complicate data integration efforts.

We emphasize the need for community awareness regarding data usage, standardized licensing practices, and associated rights as follows:

- FAIR Data: FAIR data principles (Findable, Accessible, Interoperable, Reusable) provide guidelines for structuring and documenting data to make it easily usable by others. [9]
- Clear Data Licensing Definitions: a data license acts as a legal framework to ensure data are used appropriately while adhering to the FAIR standards. Transparent and easily understandable licenses for all chemistry data are required, with the ideal being fully open data that can be used without restriction.
- Improved Provenance Tracking: The importance of documenting the origin of data to enable verification, attribution, and quality assessment should be noted.

Addressing these challenges requires collaboration among database providers, researchers, funding agencies, and publishers to establish clear guidelines, tools, and community practices for responsible data sharing and use.

Pillar 3: Coordinated community approaches regarding structure formats, ontologies, and quality curation procedures to ensure accurate association of chemical substances with associated identifiers, including structures, chemical names, and CAS Registry Numbers^®^ (CAS RNs).

Whereas public chemistry databases are vital resources, the quality and accuracy of the data they contain varies considerably. Scientists across industry, academia and government often place trust in these databases without independent validation. Errors occur across the data spectrum, starting with inaccurate, inconsistent chemical identifiers (e.g., CAS RNs, names, structures), as well as incorrect linkages of chemical identifiers to associated data, the latter spanning measured physicochemical and biological data.

The standardization of chemical representations is challenging as different databases and software tools often use different conventions for representing chemical structures, introducing inconsistencies and errors on data import/export. There have been standardization efforts by some of the largest databases, including PubChem [10], ChemSpider [11], and ChEMBL [12], but inconsistencies remain. We particularly emphasize the importance of standardized and accurate reporting of stereochemistry, such as relative and absolute designations, as errors in stereochemical representation can significantly impact accurate reporting of biological activity and other properties.

The challenges of chemical data curation are multifaceted, demanding innovative solutions and collaborative efforts. The sheer volume of chemical data available necessitates automated curation processes to as high an extent as possible. Manual curation of large datasets such as PubChem, containing tens of millions of chemicals, is clearly impractical. Hence, labor-intensive manual curation should be focused on areas of greatest error frequency (e.g., correct CAS RN-structure associations, stereochemistry) and impact (i.e., when chemical inventories are associated with regulatory standards or experimental data). Curation efforts must ensure consistency among various structure formats (e.g., InChIs, SMILES, molfiles) and specific challenges associated with conversions across software platforms, and between formats. Identifying errors requires sophisticated algorithms and tools and, whereas simple checks like charge balance can be automated, more subtle errors pertaining, for example, to tautomeric representations or relative vs. absolute stereochemistry, are difficult to detect and correct without human expertise. Curators often face the challenge of determining the most reliable source when encountering discrepancies, and resolving conflicts among different data sources can involve consulting the primary literature or commercial databases such as those from Chemical Abstracts Services. These efforts can be both time-consuming and costly, both financially and with respect to human resources [3].

Addressing errors in legacy data presents significant challenges as curation is exacting and time-consuming and there is an enormous volume of data to review. Many databases with aggregated data from primary or secondary sources incorporate pre-existing errors that have propagated through the literature and other resources. Detecting, correcting, and preventing further propagation of these errors requires not only collaboration between database providers and researchers, but a means for alerting both sources and users to corrections. Errors most often occur in linking chemical names, CAS RNs, and structures, and have been found to permeate even the largest online chemical suppliers [8], which introduces ambiguity into the identity of the chemical having been tested. Within the DSSTox project, incorrect association of CAS RNs with names and errors in structures are most commonly encountered for incorrect salt, complex, isomer, and stereochemistry designations, but can also include incorrect valency, or non-zero total charge when the compound should be neutral, or more serious frank errors.

Errors in structure-data associations will also propagate into computational models (e.g., QSARs, pharmacophore models, docking experiments, etc.) potentially leading to misleading virtual screening results and hindering chemical design and drug discovery efforts. Manual inspection of structures and comparison with other sources has been found to be essential [13, 14], but automated tools and workflows are essential for augmenting limited manual curation capabilities in processing large datasets.

Whereas structure-identifier mapping accuracy is critical for data quality, just as important is access to downloadable data for reuse. Data suppliers would ideally provide users with the ability to download relevant data for a given property in a single easily digestible download format that does not require specialized software or expertise to interpret. Ideally a downloadable dataset would include the chemical identifiers from the original source, property value qualifiers, property values, units, experimental conditions, experimental method used, and complete source metadata (the public source, the original public source, the literature source, URLs or DOIs, etc. Ideally, agreed-upon community standards would be established to allow for data download and interchange between data sources, using community ontologies if possible.

Curation efforts require expertise in both chemistry and cheminformatics. Training curators with the necessary skills and knowledge is essential for ensuring effective data curation. Promoting community engagement and collaboration is critical for improving data quality, as are quality metrics conveying the level of curation applied to online data records. The success of crowdsourced curation initiatives, such as implemented in ChemSpider, and error reporting tools and quality levels, such as provided in EPA’s Dashboard, demonstrate the potential of leveraging the collective expertise of the scientific community.

## Conclusions

Online chemistry databases are indispensable tools for researchers across diverse scientific disciplines. These databases have revolutionized access to chemical information, democratizing knowledge that was previously confined to expensive commercial platforms. New approaches to the sharing of chemical data include free data repositories including Zenodo [15] and Figshare [16] which are valuable data archiving and sharing platforms and can ensure the availability and longevity of datasets. Example chemistry datasets on these platforms include PubChemLite for Exposomics [17] on Zenodo and the EPA’s DSSTox database on FigShare [18].

The open and freely accessible nature of these resources has fostered collaboration, accelerated research, and empowered scientists worldwide with the tools to address pressing challenges in areas like drug discovery, toxicology, and environmental science. Whereas online chemistry databases offer undeniable benefits to the scientific community, their integrity and long-term utility hinges on collective and focused curation efforts to address the persistent challenges of ensuring data accuracy. We are optimistic about the future as initiatives and technological advancements, such as increased adoption of the Internal Chemical Identifier (InChI) [19], hold promise for improving the quality and reliability of these essential resources. However, we are concerned by the rise of large machine-learning and AI methods [20] that aggregate error-prone Internet resources and, can themselves, increasingly propagate errors into models. Our concern is balanced by optimism that such approaches can potentially lead to improvements in data quality [21]. Only by embracing a culture of data integrity, enforcing data reporting standards, advocating for rigorous curation practices, and fostering collaboration across the scientific community, can we ensure that online chemistry databases continue to serve as powerful engines of scientific discovery and innovation. New government-funded open data initiatives are already coming online to advance open science and encourage collaboration in chemistry. For example, the National Research Data Infrastructure (NFDI) initiative’s NFDI4Chem [22] provides a sustainable research data management infrastructure and operates in full agreement with the FAIR data principles. Such efforts are likely to expand in the future to the benefit of the chemistry community.

## Data Availability

No datasets were generated or analysed during the current study.

## References

[1] O’Boyle NM, Guha R, Willighagen EL et al (2011) Open data, open source and open standards in chemistry: the blue obelisk 5 years on. J Cheminform 3:37 21999342 10.1186/1758-2946-3-37 PMC3205042

[2] Pence HE, Williams AJ (2010) ChemSpider: an online chemical information resource. J Chem Educ 87(11):1123–1124

[3] Grulke CM, Williams AJ, Thillanadarajah I, Richard AM (2019) EPA’s DSSTox database: history of development of a curated chemistry resource supporting computational toxicology research. Comput Toxicol 12:1000996 10.1016/j.comtox.2019.100096 PMC7787967 33426407

[4] Williams AJ, Grulke CM, Edwards J et al (2017) The CompTox Chemistry Dashboard: a community data resource for environmental chemistry. J Cheminform 9:61 29185060 10.1186/s13321-017-0247-6 PMC5705535

[5] PubChem. https://pubchem.ncbi.nlm.nih.gov/. Accessed 30 Nov 2024

[6] ChEMBL. https://www.ebi.ac.uk/chembl/. Accessed 30 Nov 2024

[7] Richard AM, Judson RS, Houck KA et al (2016) ToxCast chemical landscape: paving the road to 21st century toxicology. Chem Res Tox 29(8):1225–1251 10.1021/acs.chemrestox.6b00135 27367298

[8] Richard AM, Huang R, Waidyanatha S et al (2021) The Tox21 10K compound library: collaborative chemistry advancing toxicology. Chem Res Tox 34(2):189–216 10.1021/acs.chemrestox.0c00264 PMC7887805 33140634

[9] Wilkinson M, Dumontier M, Aalbersberg I et al (2016) The FAIR guiding principles for scientific data management and stewardship. Sci Data 3:160018 26978244 10.1038/sdata.2016.18 PMC4792175

[10] Hähnke VD, Kim S, Bolton EE (2018) PubChem chemical structure standardization. J Cheminform 10:36 30097821 10.1186/s13321-018-0293-8 PMC6086778

[11] Karapetyan K, Batchelor C, Sharpe D et al (2015) The chemical validation and standardization Platform (CVSP): large-scale automated validation of chemical structure datasets. J Cheminform 7:30 26155308 10.1186/s13321-015-0072-8 PMC4494041

[12] Bento AP, Hersey A, Félix E et al (2020) An open-source chemical structure curation pipeline using RDKit. J Cheminform 12:51 33431044 10.1186/s13321-020-00456-1 PMC7458899

[13] Fourches D, Muratov E, Tropsha A (2010) Trust, but verify: on the importance of chemical structure curation in cheminformatics and QSAR modeling research. J Chem Inf Model 50(7):1189–1204 20572635 10.1021/ci100176x PMC2989419

[14] Fourches D, Muratov E, Tropsha A (2016) Trust, but verify II: a practical guide to chemogenomics data curation. J Chem Inf Model 56(7):1243–1252 27280890 10.1021/acs.jcim.6b00129 PMC5657146

[15] Zenodo. https://zenodo.org/. Accessed 20 Feb 2025

[16] FigShare. Accessed 20 Feb 2025

[17] PubChemLite for Exposomics on Zenodo. 10.5281/zenodo.4183801. Accessed 20 Feb 2025

[18] EPA DSSTox database on EPA’s FigShare. 10.23645/epacomptox.5588566.v7. Accessed 20 Feb 2025

[19] Goodman JM, Pletnev I, Thiessen P et al (2021) InChI version 1.06: now more than 99.99% reliable. J Cheminform 13:40 34030732 10.1186/s13321-021-00517-z PMC8147039

[20] Brinkhaus HO, Rajan K, Schaub J, Zielesny A, Steinbeck C (2023) Open data and algorithms for open science in AI-driven molecular informatics. Curr Opin Struct Biol 79:102542 36805192 10.1016/j.sbi.2023.102542

[21] Azeroual O (2024) Can generative ai transform data quality? A critical discussion of ChatGPT’s capabilities. Acad Engin. 10.20935/AcadEng7407

[22] NFDI4Chem. https://www.nfdi4chem.de/. Accessed 20 Feb 2025

